# Early Psychiatric Manifestations of Lafora Disease: A Case Report

**DOI:** 10.7759/cureus.111474

**Published:** 2026-06-25

**Authors:** Anouar Kaddaf, Amal Satte, Ahmed Bourazza, Mohamed Kadiri

**Affiliations:** 1 Department of Psychiatry, Mohamed V Military Teaching Hospital, Mohamed V University, Rabat, MAR; 2 Department of Neurology, Mohamed V Military Teaching Hospital, Mohamed V University, Rabat, MAR

**Keywords:** depressive disorder, epilepsy, lafora disease, myoclonic, neurodegeneration, psychotic disorders, visual hallucinations

## Abstract

Lafora disease is a rare genetic progressive myoclonic epilepsy characterized by the onset of epileptic seizures and myoclonus during adolescence, followed by a rapid progression toward neurodegeneration. However, an often underrecognized aspect of this condition is the presence of psychiatric manifestations, particularly depressive and psychotic disorders, which may emerge prior to or concurrently with the neurological symptoms.

We report the case of a 39-year-old woman with biopsy-confirmed Lafora disease whose psychiatric symptoms preceded the onset of neurological manifestations by several years. Between the ages of 12 and 16 years, she presented with recurrent suicidal behavior, episodes of wandering, severe depressive symptoms, and psychotic manifestations, including auditory and visual hallucinations, persecutory delusions, and behavioral disturbances, initially leading to psychiatric diagnoses and treatment. Neurological symptoms appeared later at the age of 20 years, with visual hallucinations followed by generalized tonic-clonic seizures, progressive myoclonus, and cognitive decline. The diagnosis of Lafora disease was ultimately confirmed by axillary skin biopsy, demonstrating periodic acid-Schiff (PAS)-positive Lafora bodies.

This case highlights the importance of a detailed investigation into the early presentation and longitudinal evolution of these psychiatric manifestations in order to distinguish them from primary psychiatric disorders and to consider them as potential clinical clues that may precede the neurological manifestations of Lafora disease. Recognition of these manifestations may facilitate consideration of an earlier diagnosis of Lafora disease and help reduce diagnostic delay in adolescents presenting with atypical psychiatric symptoms.

Furthermore, this work stresses the importance of structured interdisciplinary collaboration between neurologists and psychiatrists in the management of progressive myoclonic epilepsies and the need to develop and implement standardized recommendations for psychiatric screening in at-risk adolescents.

## Introduction

Lafora disease is a rare and severe progressive myoclonic epilepsy. First described in 1911 by the neuropathologist Gonzalo Lafora, who observed characteristic polyglucosan inclusions (Lafora bodies) in the brains of patients with progressive dementia and epilepsy. Lafora disease is an autosomal recessive genetic disorder caused mostly by mutations in two major genes: EPM2A, located on chromosome 6q24, encoding laforin, and NHLRC1, located on chromosome 6p22.3, encoding malin [1]. These mutations lead to the formation of Lafora bodies in neurones and other tissues [2], thus triggering neuroinflammatory processes [3-5].

Lafora disease is the most frequent form of progressive myoclonic epilepsy in North Africa. It generally begins between the ages of 12 and 18 years with focal occipital seizures characterized by elementary visual hallucinations, transient visual loss, or visual distortions, often followed by generalized tonic-clonic seizures and myoclonic manifestations. Subsequently, a rapid cognitive decline develops, leading to neurodegeneration and death within less than 10 years from disease onset [5, 6].

The first features of Lafora disease are heterogeneous, complex, and not pathognomonic; they often cause diagnostic delay. The diagnosis of Lafora disease is currently confirmed by genetic testing identifying pathogenic variants in EPM2A or NHLRC1. Skin biopsy, most commonly obtained from the axillary region, may demonstrate periodic acid-Schiff (PAS)-positive polyglucosan inclusions (Lafora bodies) and remains a useful complementary diagnostic tool, particularly when genetic testing is unavailable or inconclusive [7].

However, one aspect that is often overlooked is the psychiatric manifestations of the disease, which may predate or accompany the neurological symptoms, thus adding another layer of complexity to the diagnostic process. In addition to the progressive decline of cognition, the affected individuals may present severe depressive episodes and psychotic symptoms such as delusions and hallucinations [8, 9]. Such manifestations might be important early clinical clues to the disease [3, 5]. These manifestations have important implications for the timing of diagnostics and family genetic counselling.

## Case presentation

The patient was a 39-year-old single woman with no children, born to first-cousin consanguineous parents, with no significant medical history. The most remarkable aspect of this case lies in the patient’s psychiatric history, which began several years before the onset of the neurological disease.

The earliest psychiatric manifestations appeared between the ages of 12 and 15 years, during which the patient exhibited multiple suicide attempts, including bleach ingestion and medication overdoses, associated with repeated episodes of running away from home without a clear purpose or destination, lasting approximately three to four days. During this period, the family reported that she had been prescribed fluoxetine 20 mg/day by a child psychiatrist; however, treatment adherence was poor, and psychiatric follow-up was subsequently discontinued.

At the age of 16, the patient set fire to the family home. A diagnosis of brief psychotic disorder was retained, characterized by auditory and visual hallucinations, persecutory ideas directed toward family members, and the delusional belief that her parents had killed her imaginary baby. This episode improved favorably under antipsychotic treatment with olanzapine 10 mg/day. During the subsequent years, her psychiatric condition remained generally stable under treatment with olanzapine (5 mg/day). Following the discontinuation of olanzapine, neurological symptoms gradually emerged.

**Figure 1:**
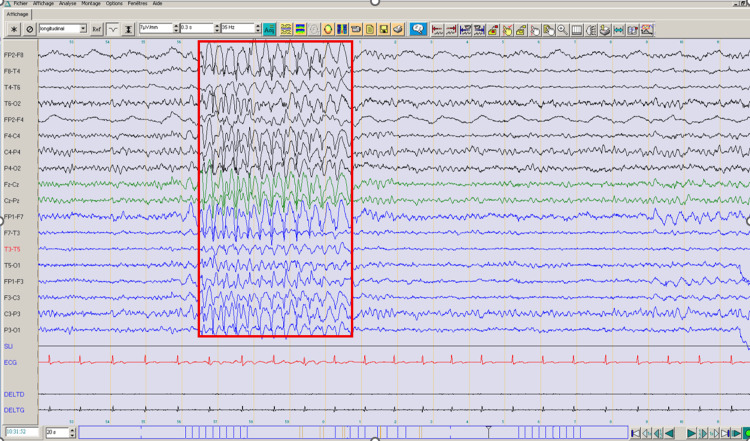
Electroencephalography: Background activity shows a slowed posterior-dominant rhythm at 8 Hz, which is abnormal for age. A well-demarcated generalized discharge is captured with an abrupt onset and offset, consisting of polyspike and slow-wave complexes at 2.5–3 Hz without recruiting rhythm (red square).

At the age of 20, she was admitted following the onset of recurrent seizures, initially characterized by focal aware occipital seizures associated with recurrent visual phenomena consisting of visual hallucinations, which subsequently evolved into focal to bilateral tonic-clonic seizures. Over time, she developed progressive myoclonus, and neurological examination revealed cognitive decline. Electroencephalographic studies revealed abnormalities compatible with progressive myoclonic epilepsy (Figure 1), while routine laboratory investigations performed during the diagnostic workup were within normal limits, including complete blood count, serum electrolytes, blood glucose, renal and liver function tests, inflammatory markers, and thyroid function. These results excluded metabolic and secondary causes of epilepsy.

**Figure 2:**
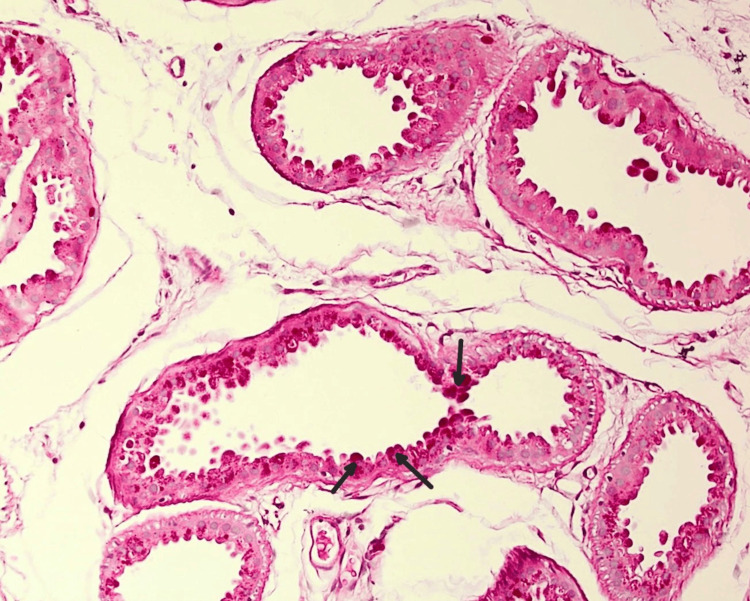
Periodic acid–Schiff (PAS) staining revealed Lafora bodies (black arrows), which are intracellular polyglucosan inclusions, in the apocrine glands of an axillary skin biopsy specimen (original magnification ×400).

Based on the combination of focal occipital seizures with visual hallucinations, progressive myoclonus, cognitive decline, parental consanguinity, characteristic EEG abnormalities, and the exclusion of alternative metabolic and secondary etiologies, Lafora disease was strongly suspected. The diagnosis was confirmed by an axillary skin biopsy, which demonstrated characteristic Lafora bodies within the cytoplasm of apocrine gland cells, appearing as rounded PAS-positive inclusions of varying sizes (Figure 2).

Genetic testing was not available at our institution at the time of diagnosis, and therefore, could not be performed.

Several antiepileptic treatments were introduced during follow-up, with partial seizure control eventually achieved using sodium valproate (1 g/day) and clobazam (10 mg/day). The patient's neurological condition subsequently remained relatively stable for several years under this treatment regimen. However, her psychiatric course continued to be marked by recurrent episodes of affective and psychotic symptoms.

In 2020, at the age of 33, during the COVID-19 lockdown period, she attempted suicide by ingesting 30 tablets of clobazam 10 mg. Following medical stabilization in the emergency department, psychiatric evaluation revealed soliloquy, auditory hallucinations, persecutory and demonic possession delusions, and a severe depressive syndrome. A diagnosis of major depressive episode with mood-incongruent psychotic features was established. Clinical evolution was favorable following treatment with sertraline 50 mg/day and risperidone initially introduced at 3 mg/day.

At present, nearly 19 years after the onset of neurological symptoms, the patient remains clinically stable, with no recurrence of psychotic symptoms or suicidal behavior and partial seizure control under low-dose risperidone (1 mg/day), sodium valproate (1 g/day), and clobazam (10 mg/day). Chronological timeline of psychiatric, neurological, diagnostic, and therapeutic events is presented in Table 1.

**Table 1:** Chronological timeline of psychiatric, neurological, diagnostic, and therapeutic events.

Age (years)	Clinical events	Diagnostic and therapeutic interventions
12–15	Recurrent suicidal behavior (bleach ingestion and medication overdoses); episodes of wandering lasting three to four days; behavioral disturbances.	Evaluated by a child psychiatrist, fluoxetine 20 mg/day was prescribed, with poor adherence and discontinuation of follow-up.
16	Brief psychotic episode characterized by auditory and visual hallucinations, persecutory delusions (belief that her parents had killed her imaginary baby), and arson of the family home.	Diagnosis of brief psychotic disorder; favorable response to olanzapine 10 mg/day.
20	Onset of neurological disease with focal occipital seizures presenting as visual hallucinations, evolving to bilateral tonic-clonic seizures.	Neurological assessment initiated.
20–25	Progressive appearance of myoclonus and cognitive decline.	EEG findings compatible with progressive myoclonic epilepsy.
During diagnostic workup	Persistent seizures and neuropsychiatric manifestations.	Routine laboratory investigations normal, excluding metabolic, infectious, endocrine, hepatic, and renal causes.
Following years	Progressive neurological evolution consistent with biopsy-confirmed Lafora disease.	Axillary skin biopsy demonstrating periodic acid–Schiff (PAS)-positive Lafora bodies, confirming the diagnosis.
Long-term follow-up	Partial seizure control	Treatment with sodium valproate 1 g/day and clobazam 10 mg/day.
2020 (age 33)	Suicide attempt by ingestion of 30 tablets of clobazam; severe depressive episode with psychotic features, including auditory hallucinations and persecutory delusions.	Emergency medical stabilization; diagnosis of major depressive episode with mood-incongruent psychotic features; treatment with sertraline 50 mg/day and risperidone 3 mg/day.
Current status (age 39)	Neuropsychiatric stability with prolonged survival despite a 19-year neurological disease course.	Maintenance treatment with risperidone 1 mg/day and antiepileptic therapy.

## Discussion

Although epilepsy and cognitive decline are the most widely recognized manifestations of Lafora disease, affected patients frequently exhibit a broad spectrum of psychiatric disturbances, often emerging at an early stage of the illness, particularly psychotic disorders and depressive syndromes [10]. Indeed, accumulation of Lafora bodies within limbic and cortical structures may contribute to the early emergence of psychiatric symptoms. Such manifestations have been reported to precede the onset of motor symptoms in some patients, making their recognition particularly important when evaluating atypical psychiatric presentations. Careful characterization of the initial psychiatric presentation and its longitudinal course is therefore essential in order to differentiate these symptoms from primary psychiatric disorders and to consider them as potential clinical clues that may precede the neurological manifestations of Lafora disease [11-12].

Depressive disorders

Severe depressive symptoms are commonly associated with marked cognitive deterioration and may occur either during the prodromal phase or throughout the progression of Lafora disease [13]. Similar findings have been reported in studies investigating early-onset dementias, suggesting the possibility of shared pathogenic mechanisms or overlapping neurobiological vulnerability. Indeed, on a cellular level, Lafora disease is characterized by the accumulation of polyglucosan inclusions (Lafora bodies) in neurons. Such deposits interfere with neuronal function, provoke inflammation, and result in neurodegeneration, especially in cortical and limbic regions involved in cognition and emotion. This may help explain the depressive and psychotic symptoms observed in some patients [14].

As observed in our patient, individuals with Lafora disease may develop major depressive episodes characterized by persistent low mood, anhedonia, sleep and appetite disturbances, psychotic symptoms that may or may not be mood-congruent, and suicidal ideation with a substantial risk of suicidal behavior.

These depressive presentations are frequently atypical and clinically complex; however, they lack distinctive features that would clearly differentiate them from depressive syndromes encountered in other rare neurological disorders [15-16].

A major clinical challenge, therefore, lies in distinguishing primary depressive disorders from depressive manifestations secondary to progressive neurological dysfunction. This distinction requires careful analysis of the clinical history, particularly family history, age at symptom onset, persistence of psychiatric manifestations, and resistance to conventional psychotropic treatment [12]. In contrast, our patient responded well to antipsychotic treatment with improvement of both psychotic and affective symptoms on olanzapine and subsequently risperidone, highlighting the heterogeneity of psychiatric treatment response in Lafora disease.

Psychotic disorders

Psychotic manifestations, including delusions and hallucinations, may occur early in the course of Lafora disease and can even precede the onset of epilepsy, thereby considerably complicating the diagnostic process [11]. Visual and auditory hallucinations, in particular, are sometimes initially interpreted as evidence of a primary psychiatric disorder, which may delay recognition of the underlying neurological disease [11]. Nevertheless, the occurrence of recurrent visual hallucinations as part of focal occipital seizures in a young patient should raise suspicion for Lafora disease [3].

Further investigation of the molecular mechanisms underlying these psychiatric manifestations is needed to clarify the relationship between Lafora bodies and the neurobiological processes associated with psychosis [13, 16], while posterior cortical hypometabolism observed on fluorodeoxyglucose (FDG)-PET imaging suggests early dysfunction of cognitive and emotional neural networks [17].

Other psychiatric manifestations

Additional psychiatric manifestations, although less frequently reported, may include behavioral disturbances such as aggression, irritability, and personality or character changes. These symptoms may arise at different stages of disease progression. Consistent with these observations, our patient exhibited significant behavioral disturbances, including repeated episodes of wandering and setting fire to the family home, several years before the onset of neurological manifestations. In many cases, relatives may misinterpret such behaviors as simple conduct problems, thereby delaying appropriate diagnosis and management [12].

Recognition of these behavioral symptoms as potential clinical clues warranting further neurological investigation may facilitate earlier identification of Lafora disease and guide appropriate diagnostic evaluation, including genetic testing when available. Obsessive-compulsive symptoms have also been described as unusual yet potentially revealing psychiatric manifestations of Lafora disease, especially in younger patients [13].

Primary psychiatric disorders or early manifestations of Lafora disease?

Psychiatric manifestations are increasingly recognized as part of the clinical spectrum of Lafora disease. In a systematic review including 298 genetically confirmed cases, cognitive symptoms were present at disease onset in 14.5% of patients, while visual symptoms were reported in 20.1% during the disease course [5]. In addition, behavioral disturbances, depression, apathy, confusion, and psychotic symptoms have been reported, sometimes appearing early in the disease course [5]. Consequently, these observations highlight the importance of considering this neurodegenerative disorder in young patients presenting with atypical psychiatric symptoms, particularly when associated with behavioral disturbances, cognitive decline, epilepsy, or an unusual clinical course. A systematic and comprehensive evaluation should therefore be undertaken to exclude underlying neurological causes. Furthermore, the broad heterogeneity of psychiatric manifestations requires rigorous clinical assessment in order to avoid misdiagnosis as primary psychiatric disorders, particularly schizophrenia, bipolar disorder, or major depressive disorder [18].

In addition to its unusual psychiatric presentation, our case is remarkable because of its prolonged survival. Classical Lafora disease is typically associated with rapid neurological deterioration and death within approximately 10 years of symptom onset. In contrast, our patient remains alive and clinically stable nearly 19 years after the onset of neurological symptoms. Although uncommon, prolonged survival has been reported in a limited number of patients and may reflect phenotypic variability related to the underlying genetic defect, modifying genetic factors, environmental influences, or advances in symptomatic management.

The differential diagnosis of Lafora disease may be particularly challenging during the early stages when psychiatric manifestations predominate. Bipolar disorder is typically characterized by cyclical mood episodes and can generally be distinguished by the absence of myoclonus and a normal EEG. Early-onset schizophrenia usually presents with positive and negative psychotic symptoms without the rapid cognitive decline that characterizes Lafora disease; brain MRI findings may be normal during the early stages of both conditions and therefore have limited value in the initial differential diagnosis. Similarly, depressive disorder is most often associated with isolated depressive episodes that respond to antidepressant treatment and are not accompanied by progressive cognitive deterioration. These clinical and paraclinical features may assist clinicians in differentiating primary psychiatric disorders from the early neuropsychiatric manifestations of Lafora disease [18].

Management recommendations

We suggest that the clinical approach should begin with a complete evaluation of any adolescent with unusual, atypical, or treatment-resistant psychiatric symptoms. This should be followed by systematic neurologic screening, including EEG, assessment for myoclonus, and detailed cognitive testing. The diagnosis should be confirmed by genetic testing whenever available. Skin biopsy may provide supportive evidence in selected cases when genetic testing is unavailable or inconclusive. Finally, optimal patient care demands a multidisciplinary approach with close collaboration among neurologists, psychiatrists, and clinical geneticists.

## Conclusions

This case emphasizes the need to keep Lafora disease in mind when evaluating adolescents or young adults who present with unusual or severe psychiatric symptoms, especially when these are accompanied by behavioral changes, psychotic features, cognitive deterioration, or epileptic manifestations. In our patient, psychiatric disturbances appeared several years before the onset of clear neurological signs and showed an initial improvement with standard psychotropic medications, a situation that may contribute to delays in reaching the correct diagnosis.

Recognition of such clinical features may prompt consideration of a more thorough neurological assessment, including electroencephalographic studies, cognitive evaluation, and genetic testing when appropriate. More broadly, this report highlights the value of close cooperation between psychiatrists and neurologists in the assessment and care of patients with Lafora disease and other progressive myoclonic epilepsies in which neuropsychiatric symptoms may play a prominent role.
